# Effect of Direct Acting Antiviral Drugs on the Occurrence and Recurrence of Intra- and Extra-Hepatic Malignancies in Patients with Chronic Hepatitis C Virus Infection

**DOI:** 10.3390/cancers16142573

**Published:** 2024-07-18

**Authors:** Pompilia Radu, Chiara Becchetti, Jonas Schropp, Patrick Schmid, Patrizia Künzler-Heule, Joachim Mertens, Darius Moradpour, Beat Müllaupt, David Semela, Francesco Negro, Markus Heim, Olivier Clerc, Maroussia Roelens, Olivia Keiser, Annalisa Berzigotti, Swiss Hepatitis C Cohort Study

**Affiliations:** 1 Department of Visceral Surgery and Medicine, Inselspital, Bern University Hospital, University of Bern, Freiburgstrasse, 3010 Bern, Switzerland; 2 Division of Infectious Diseases and Hospital Epidemiology, Cantonal Hospital St. Gallen, 9000 St. Gallen, Switzerland; 3 Division of Gastroenterology and Hepatology, Cantonal Hospital St. Gallen, 9000 St. Gallen, Switzerlanddavid.semela@kssg.ch (D.S.); 4 Gastroenterology und Hepatology, University Hospital Zürich, 8091 Zürich, Switzerland; joachim.mertens@gastrozentrum.ch (J.M.); beat.muellhaupt@usz.ch (B.M.); 5 Division of Gastroenterology and Hepatology, Lausanne University Hospital and University of Lausanne, 1011 Lausanne, Switzerland; 6 Division of Gastroenterology and Hepatology, University Hospitals Geneva, 1211 Geneva, Switzerland; 7 Division of Gastroenterology and Hepatology, University Hospital Basel, 4031 Basel, Switzerland; 8 Department of Internal Medicine and Infectious Diseases, Pourtalès Hospital, 2000 Neuchâtel, Switzerland; 9 Institute of Global Health, University of Geneva, 1205 Geneva, Switzerland

**Keywords:** direct-acting antivirals, interferon-based therapy, chronic hepatitis C, intrahepatic tumors

## Abstract

**Simple Summary:**

The introduction of direct-acting antivirals (DAAs) has significantly improved the treatment of Hepatitis C, achieving high success rates and reducing complications and deaths. Despite their success, there are concerns about the potential risk of developing liver tumors after DAA treatment. This study analyzed data from the Swiss Hepatitis C Cohort to compare the risk of liver tumors and death among patients treated with DAAs, those treated with interferon (IFN)-based therapy, and untreated patients. The findings suggest that, while DAAs reduce the risk of death and do not increase the risk of non-liver tumors, there is a higher risk of liver tumors in patients treated with DAAs compared with untreated patients. This highlights the importance of ongoing liver cancer screening for patients who have undergone DAA treatment.

**Abstract:**

**Introduction:** The use of direct-acting antivirals (DAAs) has drastically changed the management of HCV-infected patients by achieving a 95–98% sustained virologic response (SVR) and reducing morbidity and mortality in this population. However, despite their effectiveness, controversy exists concerning the occurrence of oncologic events following DAA therapy. **Aims and Methods:** A retrospective analysis was conducted on data from the Swiss Hepatitis C Cohort Study, a prospective cohort involving patients with positive HCV viremia upon inclusion, enrolled in various Swiss centers from September 2000 to November 2021. To examine potential differences in the risk of intrahepatic tumor (IHT) occurrence and death among patients treated with direct-acting antivirals (DAAs), untreated patients, and those receiving interferon (IFN)-based therapy, a semiparametric competing risk proportional hazards regression model was used. **Results:** Among 4082 patients (63.1% male, median age 45 years; genotype 1: 54.1%; cirrhosis: 16.1%), 1026 received exclusive treatment with IFN-based regimens, and 1180 were treated solely with DAAs. Over a median follow-up of 7.8 years (range: 3.8–11.9), 179 patients (4.4%) developed intrahepatic tumors (IHT), and 168 (4.1%) experienced extrahepatic tumors (EHT). The 5-year cumulative incidence of IHT was 1.55% (95% CI 0.96–2.48) for IFN-based therapy, 4.27% (95% CI 2.93–6.2) for DAA and 0.89% (95% CI 0.4–1.99) for untreated patients. There was no statistically significant difference in the risk of developing IHT (HR = 1.34; 95% CI = [0.70; 2.58]; *p* = 0.380) or death (HR = 0.66; 95% CI = [0.43; 1.03]; *p* = 0.066) between patients treated with DAAs and those treated with IFN. **Conclusions:** The DAAs reduced the risk of death and were not associated with an increased risk of extrahepatic tumors (EHT). In the adjusted model, accounting for cirrhosis and high liver stiffness, the DAA treatment was associated with a higher risk of IHT occurrence compared with untreated patients, emphasizing the relevance of implementing standardized hepatocellular carcinoma (HCC) screening post-DAA treatment.

## 1. Introduction

The advent of interferon-free antiviral regimens (direct acting antivirals (DAAs)) in the management of hepatitis C has completely changed the epidemiology of chronic liver disease of the past 10 years. DAAs allow sustained virologic response (SVR) in 95–98% of cases [1], and have determined a lower incidence of HCV-related advanced disease [2], reducing the need for liver transplantation [3] and decreasing mortality [4]. The favorable safety profile of DAAs, coupled with their efficacy, has resulted in indications for therapy extending to all infected people, with or without evident liver disease, consistent with the World Health Organization’s goal of HCV eradication by 2030 [5]. While obtaining an SVR after treatment for HCV clearly leads to a reduction in liver fibrosis [6] and portal hypertension [7], consequently reducing rates of decompensated disease [2,8] and improving survival [9], the data regarding the effect of DAAs on carcinogenesis are more controversial. Initial evidence suggested an increased recurrence rate of hepatocellular carcinoma (HCC) [10,11] following DAA treatment, decisively alerting the hepatology community. Several prospective and larger sample size studies were unable to confirm this observation [12,13,14,15], but the question about how much these drugs may act on immunomodulation [16,17,18] exists and poses an important challenge about how to adequately monitor these patients after treatment. In fact, chronic HCV infection can lead to specific genome changes, which can persist after SVR irrespective of whether it is achieved by direct antiviral agents (DAAs) or IFN-based therapies. Studies both in animal and in human cell models have demonstrated that HCV-induced epigenetic alterations are associated with HCC risk [16]. Another study has shown that hepatocytes in patients with chronic hepatitis C showed organelle abnormalities which can persist even one year after SVR. Specifically, abnormal endoplasmic reticulum was associated with patients with HCC occurring one year after SVR [19]. Indeed, on this specific focus, there is a paucity of information of long-term natural history of treated patients. Most of the available literature, in fact, deals only with the outcome of HCC recurrence in small cohorts of patients treated with DAAs, often not taking into account the complexity of a scenario that has seen, over a large time frame, changes in clinical practice and the possibility of patients undergoing multiple treatments when analyzing a short time interval.

Our study aims to evaluate whether DAA-based antiviral therapy, compared with IFN-based therapy in patients who have achieved SVR, is linked with the occurrence/recurrence of intrahepatic tumors (IHT), such as HCC, or extrahepatic tumors (EHT) in a multicentric Swiss cohort.

## 2. Methods

### 2.1. Study Population and Design

**Figure 1:**
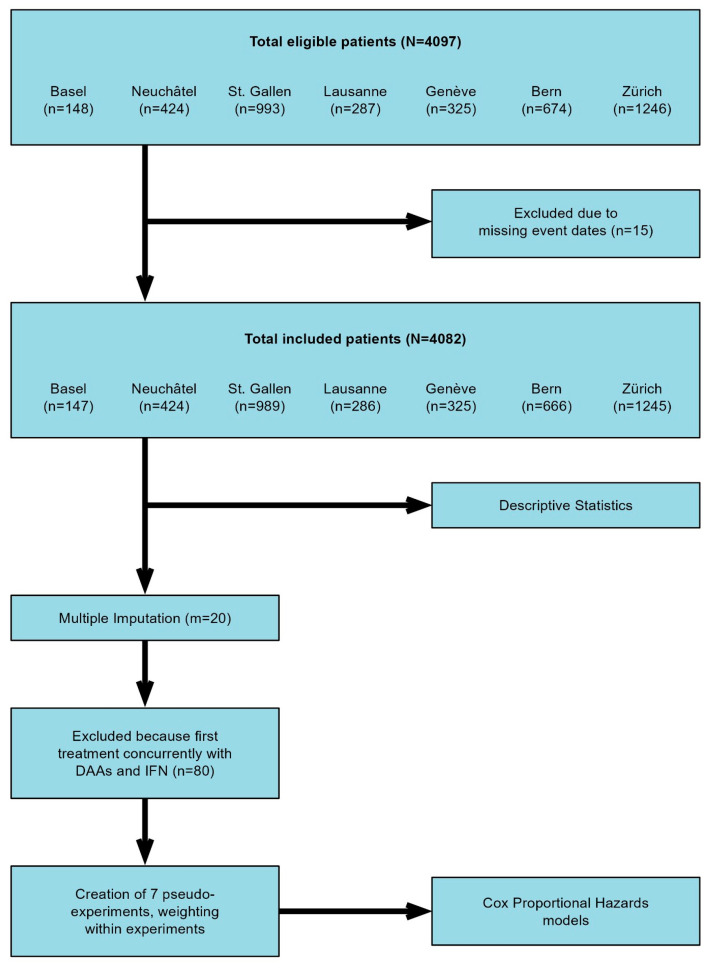
Flowchart of the study.

A retrospective analysis was performed based on data from patients enrolled in the Swiss Hepatitis C Cohort Study (SCCS), a prospective cohort consisting of patients with anti-HCV positivity and confirmed HCV viremia upon inclusion. The study comprises patients recruited at the centers in Bern, Zurich, Geneva, Lausanne, Basel, St. Gallen, and Neuchâtel, spanning the period from September 2000 and continuing until November 2021. The study was conducted in accordance with the ethical guidelines of the Declaration of Helsinki 2013 and approved by the cantonal ethics committee (KEK 2018-00727). A flowchart of the study is summarized in Figure 1. In addition to the data available from the SCCS (i.e., demographic data, HCV characteristics (e.g., genotype, SVR), stage of the liver disease and type and duration of treatments), a comprehensive review of patient files was conducted. This included the collection of information about previous history of IHT and EHT, as well as monitoring the incidence and recurrence of IHT and EHT during follow-up. The diagnosis of both IHT and E < HT was based on patients’ chart review. We define the presence of IHT and EHT when radiological or histological confirmation of neoplasia was recorded. Patients with less than 6 months follow up after inclusion in the SCCS were considered not eligible for this study.

### 2.2. Definitions

Sustained virologic response (SVR) to therapy was defined as undetectable serum HCV RNA 12–24 weeks after the cessation of treatment. Follow-up time was defined as the time from the start of treatment with DAAs, IFN-based regimens or inclusion in the SCCS cohort (for patients who were not treated) until the last visit with imaging of the liver including ultrasound, CT, or MRI, or until death. IFN-based regimens comprised pegylated- interferon (Peg-IFN) alfa 2a or alfa 2b combined with Ribavirin (RBV) for 24 weeks to 48–72 weeks. Triple therapy included Peg-IFN and RBV with the first generation of DAA (telaprevir or boceprevir). DAA regimens contained one or more of the following drugs: sofosbuvir, simeprevir, daclatasvir, ombitasvir, paritaprevir, ritonavir, dasabuvir, ledipasvir, elbasvir, grazoprevir, velpatasvir, glecaprevir, pibrentasvir and voxilaprevir [20].

### 2.3. Statistical Analysis

Quantitative variables were expressed as mean and standard deviation or median and interquartile range. Categorical variables were reported using numbers and percentages. We divided the recipients based on therapies received during the study period (i.e., DAA, IFN, both, or neither) and compared these groups using Welch’s *t*-test for continuous variables and Wilcoxon’s rank-sum test for rank data, respectively. Categorical variables were compared using Chi2. The presence of missing covariables was handled using multiple imputation by chained equations (MICE) with m = 20 imputations.

The imputation models incorporated relevant information on variables including patient sex, study center, age at enrollment, diabetes status, drinking history, patient height, weight, body mass index (BMI), HIV status, anti-HBc and HBsAg status, liver stiffness assessed by transient elastography, cirrhosis status, Child–Pugh score, history of intrahepatic tumors (IHT) or extrahepatic tumors (EHT), and platelet count. The available evidence supports employing the outcome in the process of multiple imputation for covariates [21]. Therefore, the occurrence of each examined event type during the observation time (IHT, EHT, death) as well as ln(time) until the event was used in the imputation models. To investigate whether the risk of IHT occurrence and the risk of death are different in patients treated with DAAs compared with untreated patients and patients treated with IFN-based therapy, a semiparametric competing risk proportional hazards regression model was calculated. Patients who received first-generation DAAs at the same time as IFN for their first treatment past enrollment were removed from the analysis. Additional information regarding the approach taken to manage diverse treatment regimens adopted during the follow up is provided in the Supplementary Materials.

The final model was designed in a double-robust manner, incorporating the same covariates used for weighting as predictors. Experimental allocation was modeled using strata and robust sandwich-type estimators were applied to correct standard errors for patients being part of multiple pseudo-experiments. Although the model was designed to be robust by making the groups of patients comparable (i.e., patients treated with IFN, those untreated with IFN or DAAs, and those treated with DAAs), there may still be potential missing confounders and untracked changes in covariate values after study inclusion that could affect the full extraction of the causal effects of DAA treatment.

**Figure 2:**
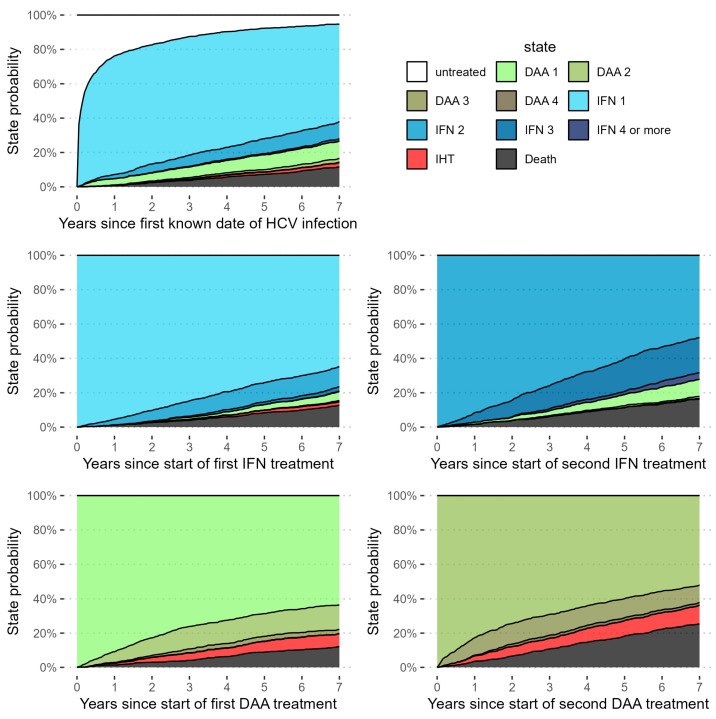
Graphical representation of the simulated state probabilities over time for a “high risk” hypothetical patient, with liver stiffness fixed at high risk (>9.6) or cirrhosis, and a BMI in the 75th quantile. It can be seen that, in this group, rates of IHT over time are likely not only higher for patients treated with DAAs, but also for those treated with repeated lines of DAAs.

Additionally, we fit several supplementary models. In the first model, we repeated the competing risks cox regression detailed above, but added EHT as an additional outcome, keeping all data preparation and analysis steps the same. Because the effects might differ for recurrence and occurrence of IHT, we fit our primary model on a subsample of the eligible patients without prior IHT. Due to the low numbers of patients with prior IHT, a model solely for this population was not feasible. We also calculated the same model in a simplified manner, without weighting on covariates, and show Kaplan–Meier curves for this model. In a model, we explored how survival and the occurrence of IHT differ in relation to the natural course of treatment. For this analysis we excluded patients who received DAA and IFN at the same time. To achieve this objective, we fit a parametric multistate survival model on each observed transition between different time points within the study, study inclusion (time 0), start of either DAAs or IFN as first therapy (time 1), subsequent therapies with either DAAs or IFN (time 2), and endpoint events such as IHT or death. Because treatment schemes throughout the study were complex and highly variable, with some patients receiving up to seven rounds of IFN during the study period, we fit this exploratory parametric multistate model to gain insight into the process of normal clinical practice and its interactions with survival and the development of IHT. In this model every stage of the treatment process, including entering the study untreated after infection, is considered a state. Patients can transition to subsequent treatment courses, as well as IHT and death, which is the only absorbing state (see Figure 2 for a graphical representation). As this last model was purely exploratory and did not take into account the number of treatment lines, it was not used for inference.

## 3. Results

### 3.1. Baseline Characteristics

**Figure 3:**
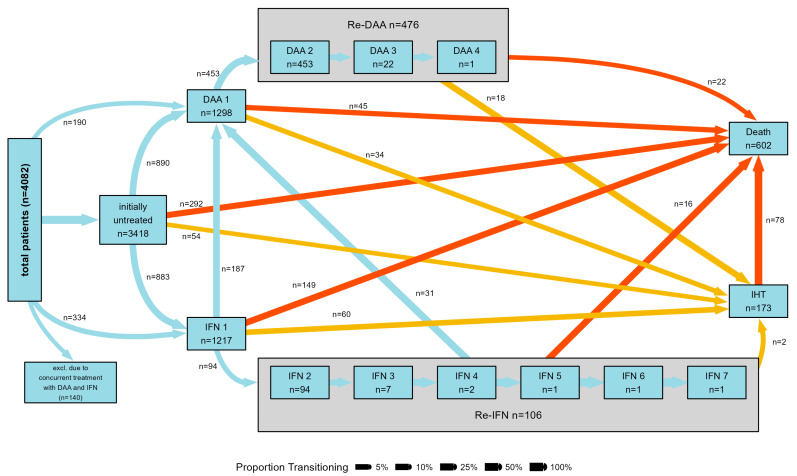
Graphical representation of the type of treatment received by patients included in the study. The figure shows patients’ transition between T0 (untreated, treated with either DAAs or IFN for the first time), repeated treatments with either DAAs or IFN and time of intrahepatic tumor diagnosis/death during the follow up. Patients who concomitantly received first-generation DAAs and IFN are excluded.

The study comprises 4082 patients and their demographic features are outlined in Table 1. The majority of participants were male (63.1%), with a median age of 45 years (range: 37–53 years), and without cirrhosis (83.6%). HCV genotype 1 prevailed among the study subjects (54.1%). Within the treated group, 1026 received exclusive IFN-based regimens, while 1180 were treated only with DAAs. SVR was reached in 650 (78%) of the patients treated exclusively with IFN-based therapy and in 933 (96%) of those exclusively treated with DAAs. Approximately 80% of patients who were non-responders, relapsers, or intolerant to IFN-based therapy underwent a subsequent cycle, involving either repeating IFN- or DAA-based regimens (Figure 3).

### 3.2. Clinical Outcome after HCV Therapy

In a median follow-up of 7.8 years (3.8–11.9), IHT developed in 179 (4.4%) patients, while EHT was detected in 168 (4.1%) patients. At the end of follow-up, 613 patients (15.3%) died. Of these, 85 had a diagnosis of IHT (13.9%) and 74 (12%) a diagnosis of EHT.

Of the 179 patients who developed intrahepatic tumors (IHT), 168 patients (93.8%) were diagnosed with HCC, 1 patient (0.6%) with hepato-cholangiocarcinoma, 8 patients (4.5%) with cholangiocarcinoma, and 1 patient (0.6%) with hepatic lymphoma. In the group of IHT, the main cause of death was liver related (progression of IHT in 59%; hepatic decompensation in 19%). At the time of IHT diagnosis, the distribution of cancer stages among patients was as follows: 9.6% were at Stage A or Stage 1, 38.5% at Stage B, 28.9% at Stage C or stage IV, and 19.2% at Stage D. Concerning the therapy assigned for IHC, in the untreated group, 11% received curative treatments, 50% underwent locoregional therapies, 33.4% received systemic treatments, and 5.6% received best supportive care. In the treated group, 29.4% received systemic treatments, 17.6% had curative treatments, 17.6% received best supportive care, and 35.3% underwent locoregional therapies. Statistical analysis showed no significant difference in survival outcomes between the treated and untreated groups (*p* = 0.346). Of the 179 patients with IHT, despite receiving maximal anti-tumor therapy, 52 patients (69%) experienced tumor progression that led to death. Additionally, 14 patients (19.5%) responded to therapy but subsequently experienced hepatic decompensation.

**Figure 4:**
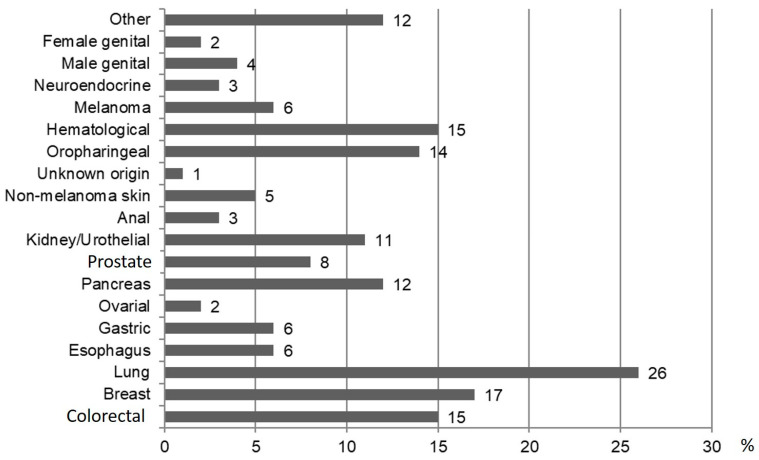
Type and frequency expressed with percentage of the EHT during the follow-up.

In the EHT group, the cause of death was tumor-related in 41 (55.4%) followed by other causes. In the IHT group, 137 were male and median age was 59 years (IQR, 53.5–67.5). Detailed characteristics are presented in Supplementary Table S1. The type of EHT diagnosed in the follow-up is shown in Figure 4. There were no significant differences regarding the occurrence of EHT, and no significant differences between patients receiving DAAs and patients receiving IFN (Supplementary Table S2A).

The 5-year cumulative incidence of IHT was 1.55% (95% CI 0.96–2.48) for IFN-based regimens, 4.27% (95% CI 2.93–6.2) for DAA and 0.89% (95% CI 0.4–1.99) for untreated patients. In patients with cirrhosis the 5-year cumulative incidence was higher, with 4.95% (95% CI 2.78–8.81), 12.45% (95% CI 8.08–19.18) and 4.54% (95% CI 1.39–14.82), respectively (Table 2). Due to the global low number of patients with a history of IHT, a separate analysis for recurrence was not performed.

### 3.3. Analysis of the Risk for IHT during Anti HCV Therapy

No significant difference in risk of IHT (HR = 1.34; 95% CI = [0.70; 2.58]; *p* = 0.380) and death (HR = 0.66; 95% CI = [0.43; 1.03]; *p* = 0.066) was found between patients receiving DAAs and those treated with IFN. However, patients assigned to DAA therapies, as first line, had a two-fold higher risk of developing IHT compared with untreated ones (HR = 2.01; 95% CI = [1.10; 3.67], *p* = 0.023) and a significant reduction in the hazard for death among patients treated with DAAs compared with those who did not receive any treatment (HR = 0.49; 95% CI = [0.33; 0.72], *p* < 0.001). Similar results were found when surveying patients if they required further courses of treatment, even if they did not switch. The hazard of IHT was increased in patients receiving DAAs compared with those receiving neither DAAs nor IFN (HR = 2.45, 95% CI = [1.29–4.83], *p* < 0.001). However, the hazard for death was lower (HR = 0.47, 95% CI = [0.30–0.72], *p* = 0.001). There was no difference compared with patients receiving IFN on this outcome (HR = 1.53, 95% CI = [0.76–3.10], *p* = 0.237). Once more, DAAs were associated with a significant reduction in the risk of death as compared with untreated patients (HR = 0.61, 95% CI = [0.37–0.99], *p* = 0.047). Regardless of whether patients were surveyed when switching to another type of treatment or when requiring a second line of treatment, the hazards for IHT were again significantly higher in patients receiving DAAs than in untreated patients, and the hazards for death were significantly lower. There were no significant differences regarding the occurrence of EHT, as well as no significant differences between patients receiving DAAs and patients receiving IFN (Supplementary Table S2A). To evaluate whether these findings might be altered by the recurrence of IHT, we excluded all patients who had a history of IHT from the data set. The models fit to this reduced sample again showed a significantly higher risk of developing IHT for patients treated with DAAs and a significantly lower risk of death compared with patients who were not treated with DAAs or IFN. There were no significant differences regarding the occurrence of EHT and no significant differences between patients receiving DAAs and patients receiving IFN (Supplementary Table S2A).

To confirm that the results were not an artifact of our method to balance the covariates, we calculated the same competing risks model without prior weighting, but still with regression adjustment for the abovementioned covariates. The results of this were found to not markedly differ from our main findings, with significantly higher risk of developing IHT in patients treated with DAAs and significantly lower hazards of death, regardless of whether we focused on the first type of treatment or the first treatment (Supplementary Table S2C). To gain insight into the process of normal clinical practice and its interactions with survival and the development of IHT, an exploratory parametric multistate model was fitted. Due to the relatively low numbers of patients receiving more than two courses of IFN-based therapy or DAA, coefficients of these transitions were constrained to represent global effects of repeated treatments. Hazards for IHT were generally constant over time regardless of the last type of treatment, with higher rates in patients receiving more than one course of DAAs. The hazards of death appeared accelerate over time when transitioning from any of the treatments, possibly due to the natural course of aging, but not for patients diagnosed with IHT, where the highest hazards of death were within five years after diagnosis.

We simulated paths through this model to estimate expected proportions of patients in each of the states after specified periods of time. Patients in this simulation would start in one of five states—untreated, treated with IFN based therapy, treated with DAAs, repeated treatment with IFN regimes and repeated treatment with DAAs—and would transition through each allowed state based on the probabilities determined from the multistate model (Figure 2).

## 4. Discussion

The findings from this multicentric study align with previous research [22,23], indicating that the use of DAAs for antiviral therapy is linked to a decreased mortality risk among HCV patients. Additionally, there was no evidence supporting an association between DAA treatment and the development of EHT. Furthermore, in terms of potential concerns about an elevated risk of IHT occurrence, our study indicates that the risk in patients treated with DAAs is similar to that seen in patients treated with IFN-based therapy. However, we found that DAAs are associated with an increased risk of IHT as compared with untreated patients with chronic hepatitis C, after balancing the data on several known risk factors and controlling for them, including presence of cirrhosis and liver stiffness. We also observed a higher risk for IHT in the DAA group compared with the IFN group in the model without covariate weighting, but this difference was not consistently seen after covariate weighting, suggesting that it is due to insufficient covariate adjustment. We confirmed these findings through a robust statistical approach simulating different scenarios and taking into account the possibility of therapy switch.

It is worth mentioning that, in our population, the annual risk of HCC was generally low (0.18–0.82% per year). Previous data have shown that, in patients treated with IFN-based therapy, the risk of HCC decreases as compared with untreated patients and, in those who achieve SVR, HCC incidence varies between 0.33% per year in patients without cirrhosis and 1.4% per year in patients with cirrhosis [24,25]. One of the possible explanations for this finding is the low proportion of patients with cirrhosis (16%) in our cohort. Indeed, in the group of patients with cirrhosis, incidence of IHT was much higher (around 1% per year in untreated patients and in patients exposed to IFN, and around 3% per year in patients exposed to DAAs), and in line with previous data. Therefore, these higher HRs for IHT and DAAs must be downsized accordingly, though they still corroborate the idea that an accurate policy of surveillance after SVR achieved with DAAs need to be implemented. The observed risk of HCC was about 1% per year in our patients undergoing therapy with DAAs in the overall group and was around 3% per year in patients with cirrhosis; incidence was roughly constant at 1, 3 and 5 years. In untreated patients with cirrhosis, the incidence of HCC is expected to be 3–5% per year. A recent study on almost 4000 subjects receiving DAAs has shown that DAA treatment in patients with compensated cirrhosis was associated with a lower risk of HCC (aHR, 0.57; 95% CI, 0.37–0.88) compared with untreated patients, but this association was not statistically significant for patients without cirrhosis or with decompensated cirrhosis [26]. The international guidelines suggest staging fibrosis before treatment, either evaluated by transient elastography or directly assessed through liver biopsy, as liver fibrosis stage is the main risk factor for HCC development in patients treated with DAAs. This prompts the implementation of HCC screening programs after SVR in patients with a fibrosis stage above F3 [20]. However, the question of how to weight other known cancerogenic co-factors, such as alcohol or smoke exposure, family history or concomitant metabolic-associated steatotic liver disease (MASLD) [15,27,28], even with fibrosis less pronounced than F3, remains open. Indeed, there is a lack of systematic data in this setting, and our study, despite its limitations, provides additional evidence on these aspects in an oncological perspective, by a wide epidemiological overview on the natural history of hepatitis C after its cure. In addition to our data, there is only a recent study, which included 185 subjects, to have prospectively addressed the incidence of HCC in patients with F3 or F4 fibrosis without previous history of liver nodules prior to DAA treatment [29]. The study found no HCC occurrence in patients with F3 (63 patients), while 10 patients with cirrhosis developed HCC. Despite this reassuring prospective data in F3 patients, the interpretation of the incidence rate of HCC in F4 should be taken with caution due to the small sample size.

Our study has the strength of being based on prospective cohorts at several centers, with adequate follow-up. In addition, the statistical analysis has been extensive and covariate balancing and regression adjustment provides robust estimates. Whether DAAs are responsible for the slightly higher rate of IHT as compared with untreated patients, or whether this could be due to other causal factors, which we could not capture in this study, remains unknown, and our study cannot provide mechanistic insight. The fact that DAAs can cause immunomodulation even after reaching SVR has been demonstrated in several studies [16,17,18,30]. Human HCC cells express vascular endothelial growth factor (VEGF), which modulates growth and survival and it has been shown that treatment with DAAs leads to a marked increase in VEGF in patients with cirrhosis (a 4-fold increase) [30]. In addition, despite SVR induced by DAA treatment significantly reducing organelle abnormalities in hepatocytes infected by HCV, this did not change the alterations in the endoplasmic reticulum, which seemed to have worsened 1 year after eradication, being associated with the development of HCC [19]. In an analysis of liver tissues from patients with and without an SVR due to DAA therapy, epigenetic and gene expression alterations associated with risk for HCC have been found [16]. Indeed, patients with small (<1 cm) HCC prior to DAA exposure might potentially face an accelerated growth and clinically evident tumor, similar to what has been shown for non-characterized liver nodules present prior to treatment [31]. It is worth mentioning that a significant proportion of patients in our cohort experienced disease progression despite therapeutic interventions.

The current study has some limitations that should be taken into account. First, patients with cirrhosis underwent regular HCC screening. This may result in an over-detection of HCC compared with EHT, which is more likely to be identified incidentally or based on clinical symptoms. This discrepancy could introduce a bias in the detection rates of IHT versus EHT. Secondly, we do not have detailed information on smoking/smoking cessation or on information on steatosis or metabolic syndrome (with the exception of diabetes), and those both are well-known risk factors for the development of HCC [28,32]. Given the small number of IHT diagnosed in the untreated patients, we cannot exclude underdiagnosis. However, patients were prospectively followed up with in the SCCS cohort, and underwent imaging as part of the SCCS study protocol, irrespective of whether they were treated or untreated. Patients treated with DAAs were followed up more closely than the other groups given the standard treatment protocols, leading to early HCC diagnosis. This might also be an explanation for the low cancer-related mortality found in this group. It is important to consider that the data collected covers a relatively long time in which the availability and prescription of non-interferon-containing regimens has changed. In fact, until 2018–2019, DAAs in Switzerland were prescribed and reimbursable only for patients with advanced fibrosis or cirrhosis, so these patients were regularly screened according to European guidelines [33]. On the other hand, patients with low fibrosis levels (F0–F1), defined by non-invasive methods or biopsy before the start of treatment, did not undergo HCC screening. However, it is worth mentioning that the follow-up data were systematically collected in the Swiss Hepatitis C Cohort Study. Unfortunately, the available data, which are not time dependent, did not allow us to consider disease progression over time as a potential confounder of the association between treatment and onset IHT and EHT. Furthermore, given the small number of patients with previous IHT, most of whom were treated with DAAs late in their history, we could not answer the question as to whether DAAs increase or decrease the risk of recurrence of this type of tumor. Lastly, several of the included patients underwent multiple treatments over time. The decision to retreat and the type of treatment largely depended on the drugs available on the market and on the hepatologist in charge. However, the statistical model adopted has the strength to have taken this aspect into consideration. Indeed, weighting was considered to balance the covariates between patients treated with DAAs and the IFN/untreated groups in order to make them more comparable, reducing the potential for biased estimates due to confounding factors and to immortal bias. In our cohort, the rate of untreated patients is relatively high (40%). This can be attributed to the delayed accessibility of direct-acting antiviral (DAA) treatment for patients without fibrosis in Switzerland compared with other European countries.

## 5. Conclusions

The results of the current study show that DAAs are associated with a lower risk of death in the population of the study. Additionally, DAA treatment is not associated with an increased risk of EHT, and the risk of IHT is comparable to that observed in patients exposed to IFN-based therapy. However, after controlling for several known risk factors (i.e., cirrhosis, high liver stiffness pre-treatment), DAA treatment was found to be associated with an increased risk of IHT as compared with untreated patients. This suggests the importance of prolonged HCC surveillance in patients with advanced liver disease/cirrhosis after DAA treatment.

## Figures and Tables

**Table 1:** Baseline characteristics of patients included in the present study.

	Overall (Based on Data Available)	Both Therapies (Combined or Sequential Use)	DAAs Only	IFN-Based Only	Untreated during FU	*p*-Value *
N	4082	357 (140 combined)	1180	1026	1519	
**Age, years**	45 [37–53]	47 [41–53]	48 [40–55]	43 [35–50]	44 [36–52]	<0.001
Gender, Male	2575 (63.1)	249 (69.7)	728 (61.7)	673 (65.6)	925 (61)	0.004
Missing data	2 (0.0)	0 (0.0)	0 (0.0)	0 (0.0)	2 (0.1)	
**BMI**	24.06 [21.71–26.79]	25.0 [22.65–27.40]	24.30 [21.8–27]	23.77 [21.51–26.56]	23.83 [21.48–26.48]	<0.001
**Genotype**						<0.001
1	1981 (48.5)	225 (63)	641 (54.3)	437 (42.6)	678 (44.6)	
2	266 (6.5)	12 (3.4)	57 (4.8)	90 (8.8)	107 (7)	
3	1003(24.6)	71 (19.9)	204 (17.3)	392 (38.2)	336 (22.1)	
4	405 (9.9)	38 (10.6)	125 (10.6)	78 (7.6)	164 (10.8)	
5	6 (0.1)	0 (0.0)	3 (0.3)	2 (0.2)	1 (0.1)	
6	3 (0.1)	0 (0.0)	1 (0.1)	2 (0.2)	0 (0.0)	
Missing data	418	11	149	25	233	
***Comorbidities***						
**Diabetes**, Yes	212 (5.2)	21 (5.9)	60 (5.1)	60 (5.8)	71 (4.7)	0.577
Missing data	18	1	6	0	11	
**Heavy Drinking**, Yes	2061 (50.5)	172 (48.2)	613 (51.9)	517 (50.4)	759 (50)	0.517
Missing data	9	1	4	0	4	
***Co-infection***						
**HIV infection** **Yes**	258 (6.9)	27 (7.8)	50 (4.8)	37 (3.8)	144 (10.6)	<0.001
Missing data	355	12	128	53	162	
**Anti-HBc** Yes	1473 (39.9)	142 (41.5)	390 (37.1)	392 (41.1)	549 (40.8)	0.175
Missing	390	15	128	73	174	
**HBsAg**, Yes	901 (24.1)	94 (27.3)	195 (18.7)	209 (21.2)	403 (29.5)	<0.001
Missing	345 (8.5)	13 (3.6)	139 (11.8)	41 (4.0)	152 (10)	
**Cirrhosis, yes**	654 (16.1)	97 (27.2)	192 (16.3)	178 (17.4)	187 (12.4)	<0.001
Missing	14	0	4	5	5	
**Liver stiffness**, **kPa**	6.60 [4.80–10.40]	8.60 [6.10–14.40]	7.00 [5.03–11]	6.20 [4.70–10.40]	5.90 [4.60–7.85]	<0.001
**Child Pugh score**	5 [5–6]	5 [5–6]	5 [5–5]	5 [5–6]	5 [5–6]	0.025
**PLT <150.000**, Yes	2527 (75.2)	210 (66)	620 (79.4)	652 (67.2)	1045 (81)	<0.001
Missing	723	39	399	56	229	
**SVR**						<0.001
Yes	1860 (76.9)	277 (92.3)	933 (96)	650 (78)		<0.001
Unknown **	458 (11.2)	57 (16)	208 (17.3)	193 (18.8)		
**History of cancer (IHT or EHT)**						
Yes	80 (3.5)	13 (6)	14 (1.8)	26 (4.5)	27 (3.7)	0.006
Missing	1781	142	401	442	796	
**Development of events in the follow-up**						
EHT (yes)	168 (4.1)	22 (6.2)	39 (3.3)	47 (4.6)	60 (3.9)	0.093
IHT (yes)	179 (4.4)	36 (10.1)	42 (3.6)	59 (5.8)	42 (2.8)	<0.001
Death (yes)	613 (15.0)	38 (10.6)	64 (5.4)	199 (19.4)	312 (20.5)	<0.001

Abbreviations: BMI, body mass index; IFN, interferon; DAA, direct antiviral agents; PLT, platelet; HBsAg, Hepatitis B surface antigen; Anti-HBc, Hepatitis B core; n, number of available patients for a specific variable. * *p*-values refer to the overall difference between the four groups (both therapies, DAAs only, IFN only, untreated) and were calculated using one-way ANOVA, Kruskall–Wallis, the Chi2-test or Fisher’s exact test. ** Includes those patients who had not finished the treatment.

**Table 2:** Cumulative incidence of IHT in the study cohort overall and considering cirrhosis.

Time	IFN-Based Regimens Cumulative Incidence (95% CI)	DAA Cumulative Incidence (95% CI)	Untreated Cumulative Incidence (95% CI)
1 year	0.17 [0.04–0.68]	0.82 [0.43–1.58]	0.18 [0.03–1.3]
3 years	0.89 [0.48–1.64]	2 [1.29–3.09]	0.65 [0.25–1.74]
5 years	1.55 [0.96–2.48]	4.27 [2.93–6.2]	0.89 [0.4–1.99]
10 years	4.78 [3.59–6.38]		2.07 [1.29–3.31]
15 years	9.02 [6.96–11.68]		3.3 [2.33–4.68]
20 years			4.94 [3.72–6.57]
**Cirrhosis**			
	**IFN-based regimens** **Cumulative Incidence (95% CI)**	**DAA** **Cumulative Incidence (95% CI)**	**Untreated** **Cumulative Incidence (95% CI)**
1 year	0.43 [0.06–3.06]	2.80 [1.18–6.65]	2.38 [0.34–16.51]
3 years	2.22 [0.93–5.28]	7.52 [4.46–12.59]	2.38 [0.34–16.51]
5 years	4.95 [2.78–8.81]	12.45 [8.08–19.18]	4.54 [1.39–14.82]

Abbreviations: DAA, direct antiviral; IFN, interferon; 95% CI, confidence interval. Cumulative incidence at 20 years for IFN and 10, 15 and 20 years for DAA not calculated due to insufficient data.

## Data Availability

The raw data supporting the conclusions of this article will be made available by the authors on request.

## Supplementary Materials

The following supporting information can be downloaded at: https://www.mdpi.com/article/10.3390/cancers16142573/s1, Table S1. General characteristics of patients developing IHT stratified for the type of antiviral treatment adopted; Table S2. The risk of IHT and EHT in patients treated with DAAs compared to untreated patients and patients treated with IFN according to different models tested.

## Abbreviation

Direct-acting antivirals—DAAs; sustained virological response—SVR; intrahepatic tumors—IHT; extrahepatic tumors—EHT; interferon—IFN; hepatitis C virus—HCV; hepatocellular carcinoma—HCC; ribavirin—RBV; Swiss Hepatitis C Cohort Study—SCCS; body mass index—BMI; metabolic-associated steatotic liver disease—MASLD; vascular endothelial growth factor—VEGF.
