# Supplementary material for: Effect of Direct Acting Antiviral Drugs on the Occurrence and Recurrence of Intra- and Extra-Hepatic Malignancies in Patients with Chronic Hepatitis C Virus Infection
Source: Cancers (Basel). 2024 Jul 18;16(14):2573. doi: 10.3390/cancers16142573 (PMC11275029; doi:10.3390/cancers16142573)
Supplement: Supplementary file 1 [file cancers-16-02573-s001.zip › cancers-3080049-supplementary.pdf]

Supplementary Materials

Statistical supplementary material

In order to be able to start the follow-up time at the time the first treatment was administered, we created seven pseudo-experiments, starting at 0, 0.5, 1, 2, 4, 8 and 16 years past the first known date of infection with HCV. Patients were then assigned into these pseudo-experiments with replacement, such that a patient could first be assigned to multiple experiments as long as they were untreated, but could only be in one experiment once they were treated with either DAAs or IFN (rolling-entry matching on coarsened time). This enabled us to incorporate the fact that patients could change treatment status during the follow up and those events could occur even before the start of treatment. In addition, it increased the number of untreated controls for each patient that was treated with DAAs. The three groups were then balanced on patient age, BMI, and sex, whether they had already been treated before enrollment into the study, HIV status, Anti-HBc, presence of cirrhosis, median liver stiffness measured by transient elastography, study center and platelet count within these strata using entropy weighting (20). The treatment effect of DAAs was used as the focal group in weighting. Patients were censored when they switched to another type of treatment (from DAAs to IFN or from IFN to DAAs), but not if they received another round of treatment of the same type in our main analysis (model a, first type of treatment). We complimented this by also censoring patients if they received another round of treatment, regardless of whether the medication used was the same (model b, first treatment). Both death and the occurrence of IHT were treated as sinking states because the number of patients developing IHT was not sufficient to estimate treatment effects for this transition.

Supplementary. Table S1. General characteristics of patients developing IHT stratified for the type of antiviral treatment adopted.

	IFN-based therapy	DAA-based therapy	Both therapies (sequential use)	Untreated
**Number of cases (% of the overall group)**	60 (34)	41 (23)	34 (19)	44 (25)
**Age**	60.0±10.1	63.0±10.5	57.27	71
**Gender (male)**	44(73.3)	30(73.2)	30(88.2)	33(75)
**Cirrhosis (Yes)**	28(46.7)	26(63.4)	21(61.8)	29(65.9)
**SVR**				
**Yes**	23(38.3)	31(75.6)	28(83.4)	NA
**No**	29(48.3)	5(12.2)	4(11.8)	NA
**Missing**	8(13.1)	5(12.2)	2(5.9)	NA
**Type of tumor**				
**Hepatocellular carcinoma (HCC)**	56(93.3)	40(97.6)	32(94.1)	41(93.2)
**Cholangiocellular carcinoma**	4(6.7)	0(0)	2(5.9)	2(4.5)
**Mixed Hepato-Cholangiocellular carcinoma**	0(0)	1(2.4)	0(0)	0(0)
**Intrahepatic Lymphoma**	0(0)	0(0)	0(0)	1(0.6)
**Tumor stage***				
**0**	6(10)	4(9.8)	3(8.8)	4 (9.1)
**BCLC stage A/I**	18(30)	23(56.1)	16(47.1)	10(22.7)
**BCLC stage B/II**	20(33.3)	4(9.8)	9(26.5)	12(27.3)
**BCLC stage C/III**	8(13.3)	4(9.8)	3(8.8)	11(25)
**BCLC stage D/IV**	5(8.3)	2(4.9)	2(5.9)	4(9.1)
**Missing**	3 (5)	4(9.8)	1(2.9)	3(6.8)

Abbreviations: SVR, sustained viral response; DAA, direct antiviral, INF, interferon, NA not applicable, *BCLC staging system was used for HCC staging, TNM staging system was used for the remaining tumors

Supplementary Table S2. The risk of IHT and EHT in patients treated with DAAs compared to untreated patients and patients treated with IFN according to different models tested.

A) Results if considering EHT as a competing risk

First Type of Treatment				
**Event**	**term**	**HR**	**95% CI**	**p**
**IHT**	DAA vs IFN	1.30	0.66-2.53	0.449
	DAA vs untreated	1.89	1.03-3.46	0.039
**EHT**	DAA vs IFN	0.82	0.42-1.6	0.561
	DAA vs untreated	1.52	0.82-2.82	0.184
**Death**	DAA vs IFN	0.77	0.48-1.24	0.285
	DAA vs untreated	0.44	0.29-0.66	0.000

First Type of Treatment				
**Event**	**term**	**HR**	**95% CI**	**p**
**IHT**	DAA vs IFN	1.5	0.76- 3.13	0.232
	DAA vs untreated	2.5	1.29-4.83	0.007
**EHT**	DAA vs IFN	0.74	0.34-1.62	0.453
	DAA vs untreated	1.25	0.62-2.51	0.535
**Death**	DAA vs IFN	0.70	0.41-1.19	0.190
	DAA vs untreated	0.43	0.27-0.68	<0.001

B) Results after excluding patients with history of IHT.

First Type of Treatment				
**Event**	**term**	**HR**	**95% CI**	**p**
**IHT**	DAA vs IFN	2.26	0.87-5.87	0.095
	DAA vs untreated	2.34	1.07-5.12	0.034
**EHT**	DAA vs IFN	0.67	0.26-1.76	0.420
	DAA vs untreated	0.81	0.34-1.89	0.618
**Death**	DAA vs IFN	0.75	0.40-1.41	0.369
	DAA vs untreated	0.40	0.23-0.7	0.001

First Type of Treatment				
**Event**	**term**	**HR**	**95% CI**	**p**
**IHT**	DAA vs IFN	1.91	0.70- 5.2	0.203
	DAA vs untreated	2.2	0.98-4.93	0.056
**EHT**	DAA vs IFN	0.74	0.25-2.16	0.546
	DAA vs untreated	0.78	0.31-1.92	0.463
**Death**	DAA vs IFN	0.82	0.42-1.59	0.338
	DAA vs untreated	0.43	0.25-0.74	0.003

C) Results of risk models without prior covariate weighting but using regression adjustment.

First type of treatment				
**Event**	**term**	**HR**	**95% CI**	**p**
**IHT**	DAA vs IFN	1.64	0.96-2.81	0.073
	DAA vs untreated	2.15	1.22-3.78	0.008
**EHT**	DAA vs IFN	1.12	0.61-2.06	0.727
	DAA vs untreated	1.18	0.66-2.12	0.574
**Death**	DAA vs IFN	0.78	0.52-1.15	0.200
	DAA vs untreated	0.48	0.36-0.70	<0.001

First type of treatment				
**Event**	**term**	**HR**	**95% CI**	**p**
**IHT**	DAA vs IFN	2.33	1.31- 4.16	0.004
	DAA vs untreated	3.08	1.69-5.62	<0.001
**EHT**	DAA vs IFN	0.91	0.44-1.87	0.797
	DAA vs untreated	0.99	0.5-1.95	0.967
**Death**	DAA vs IFN	0.67	0.43-1.04	0.073
	DAA vs untreated	0.41	0.27-0.63	<0.001

Abbreviations: IHT, intrahepatic cancer; EHT, extrahepatic cancer; DAA, direct antiviral, INF, interferon.
